# Late postpartum depression and associated factors: community-based cross-sectional study

**DOI:** 10.1186/s12905-023-02444-7

**Published:** 2023-05-23

**Authors:** Lema Fikadu Wedajo, Solomon Seyife Alemu, Mohammedamin Hajure Jarso, Aman Mamo Golge, Dejene Edosa Dirirsa

**Affiliations:** 1 Department of Midwifery, Mattu University College of medical and Health Sciences, Mattu, Ethiopia; 2 Department of Midwifery, Madda Walabu University College of Medicine and Health Sciences, Sheshemene, Ethiopia; 3 Department of Psychiatry, Walabu University College of Medicine and Health Sciences, Sheshemene, Ethiopia; 4 Department of Nursing, Madda Walabu University College of Medicine and Health Sciences, Sheshemene, Ethiopia; 5 College of Medicine and Health Sciences, Department of Midwifery, Salale University, Salale, Ethiopia

**Keywords:** Late postpartum Depression, Postpartum Mothers, Arba Minch, Ethiopia

## Abstract

**Background:**

Late postpartum depression is the presence of depressive symptoms beyond the early postpartum period and is a significant mental health problem that has a devastating impact on mothers, infants, partners, family members, the healthcare system, and the world’s economy. However, there is limited information regarding this problem in Ethiopia.

**Objective:**

To assess the prevalence of late postpartum depression and associated factors.

**Method:**

the community-based cross-sectional study was employed among 479 postpartum mothers in Arba Minch town from May 21 to June 21, 2022. The pre-tested face-to-face interviewer administered a structured questionnaire used to collect the data. A bivariate and multivariable analysis was done using a binary logistic regression model to identify factors associated with late postpartum depression. Both crude and adjusted odds ratios with 95% CI were calculated, and a p-value of < 0.05 was used to declare statistically significant factors.

**Result:**

The prevalence of late postpartum depression was 22.98% (95% CI: 19.16, 26.80). Husband Khat use (AOR = 2.64; 95% CI: 1.18, 5.91), partner dissatisfaction with the gender of the baby (AOR = 2.53; 95% CI: 1.22, 5.24), short inter-delivery interval (AOR = 6.80; 95% CI: 3.34, 13.84), difficulty to meet husband sexual need (AOR = 3.21; 95% CI: 1.62, 6.37), postpartum intimate partner violence (AOR = 4.08; 95% CI: 1.95, 8.54), and low social support (AOR = 2.50; 95% CI: 1.25, 4.50) were significantly associated factors at p-value < 0.05.

**Conclusion:**

Overall, 22.98% of mothers suffered from late postpartum depression. Therefore, based on the identified factors, the Ministry of Health, Zonal Health Departments, and other responsible agencies should establish effective strategies to overcome this problem.

## Introduction

Postpartum depression (PPD) is a treatable non-psychotic mental disorder that is manifested by depressive symptoms like mood fluctuation, loss of happiness, diminished activity, functional impairment, low self-esteem, and even attempts at suicidal behavior [1–3]. The problem is common during the first six weeks after childbirth and can persist for up to a year after childbirth if not treated [4]. The exact cause of PPD is not clear, but scholars have identified: life stress, depression during pregnancy, withdrawal of hormones, being a mother to a new baby, thinking about her and her newborn baby as well as her partner, poor social support, and low socio-economic status [5–7].

Globally, PPD affects around 18% of mothers [8, 9], and a systematic analysis of studies carried out in East Africa from 1998 to 2018 revealed that 24% of breastfeeding mothers suffered from it [10], In sub-Saharan Africa, 18.6% [11], and in Ethiopia, 12.2–33% of postpartum mothers were the victims [12].

Postpartum depression affects mothers and newborns through impaired mother-infant bonding, child abuse, child neglect, early discontinuation of breastfeeding, and decreased nutrition intake that leads to some morbid conditions like poor weight gain, anemia, and hypertension [13, 14]. If untreated, it leads to substance abuse, self-harm, and attempts at suicidal behavior and infanticide [15], and around 22% of maternal deaths are due to LPPD [16]. The consequences of postpartum depression are wide and affect the health of the mother, newborn, male partners, and other family members as a whole [17–20], as well as exclusive breastfeeding, immunizations, and complementary feeding practices [11, 21–24].

Studies indicate that 4–25% of fathers can experience symptoms of postpartum depression as a result of maternal depression [25, 26], which results in divorce, custody disputes, and loneliness [27]. Furthermore, LPPD can impair the cognitive development of the new generations [28, 29]. In addition, LPPD has a great impact on economies through the time and money spent on treatment as well as the productivity of mothers as indicated by the study conducted in Texas, where maternal mental health conditions affect 17.2% of mothers and cost $962 million per year [30].

The United Nations Sustainable Development Goal has included “mental health and health for all” in its agenda [31]. The global community’s experts agreed on strategies, among which, by 2030, Goal 3 states that health for all as it is one domain of maternal and child health; that might include maternal mental health [32]. Late postpartum depression (LPPD) is a late-onset depression that happened a few weeks after delivery to twelve months of the postnatal period [33].

It is a neglected problem that endangers maternal health during late postpartum period. The reason is that during this period there was no screening of mothers for depression as that of early postpartum period because there is a no regular checkup during this time for mental health problem. Thus, mothers need special attention during the late postpartum period to maintain their health. In Ethiopia, there is a pocket of research on late postpartum depression, even if it has devastating multidimensional health consequences. Therefore, this study aimed to assess the prevalence of late postpartum depression and associated factors in Arba Minch Town. The finding of this study may help the government and ministry of health to develop effective strategy; and enable health care professionals to provide evidence-based care plan to overcome this problem.

## Method

### Study design, area and period

A community-based cross-sectional study design was carried out from May 21 to June 21, 2022, in Arba Minch town, Gamo Zone, in the southern part of Ethiopia. Arba Minch town is located 505 km south of Addis Ababa, the capital city of Ethiopia, and is the administrative center of the Gamo zone in the South Nation Nationalities Peoples of Ethiopia. Four governmental health institutions, namely Arba Minch General Hospital, Dilfana Primary Hospital, Shecha Health Center, and Woze Health Center, along with other private health institutions, are serving Arba Minch town as well as the surrounding populations. The town has twelve kebele with 24,090 households and 112,724 people living in the town. The expected deliveries in Arba Minch town are 4,272 annually [34]. All the government health institutions in this town have functional maternity and child health care settings that provide care for the community of Arba Minch town and Arba Minch area with five obstetricians and gynecologists, eighty-eight midwives, and nine general practitioners at government health institutions in this town.

### Source Population

All postpartum mothers who were in the first year after childbirth in the twelve kebele of Arba Minch town.

### Study population

All postpartum mothers who were in the first year after childbirth and residents of Arba Minch town for at least six months during the data collection period.

### Inclusion and exclusion criteria

All postpartum mothers who were in the first year after childbirth during the data collection period and were ≥ 18 years old were included in the study. Critically ill mothers during the data collection period who were in pain and unable to respond and those who were in the first six weeks of the postpartum period were excluded. In addition, mothers with known or diagnosed mental health problems were excluded from this study.

### Sample size determination

The sample size was calculated by using a single population proportion formula based on the proportion of late postpartum depression, 23.7%, which was conducted in Awi Zone, Ethiopia [35]. It was calculated based on assumptions of a 95% confidence level with 1.96 degrees of precision and a margin of error of 4% based on the rule of thumb as follows:

$$\text{n} =\frac{(z\alpha /2)2\left(pq\right)}{d2 }, \text{n}=\frac{\left(1.96\right)2\left(0.237???0.763\right)}{\left(0.04\right)2}; \text{n}= 435.$$

Where, n = sample size.

p = proportion of previous study.

$z\alpha /2$ = degree of precision

d = margin of error.

Finally, by adding 10% of the non-response rate to the final sample size, 479 mothers were taken for the purpose of this study.

### Sampling techniques

**Fig. 1:**
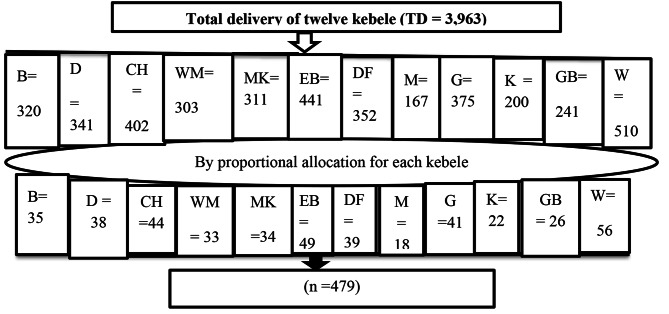
Schematic presentation of sampling procedure to assess the prevalence of late postpartum depression in Arba Minch town, Gamo zone, Southern Ethiopia, 2022 **Footnote**: TD – Total delivery, B - Bere, D – Doysa, CH – Chamo, WM – Wuha Minch, MK = Mehal Ketema, EB – Edgetber, DF – Dilfana, M – Menarhea G = Gurba, K – Kulfo, GB – Gedib, W – Woze, n – final sample size

Initially, the total number of mothers who were in the first year after childbirth in the twelve kebele of Arba Minch town was obtained from the health extension workers’ log books. Based on the obtained data, the total number of postpartum mothers from each kebele was proportionally allocated to each kebele to get the final sample size. Then, the obtained number of postpartum mothers in each kebele was converted to table random numbers based on the family folder code given by Health Extension Workers, and by computer-generated simple random sampling techniques, eligible mothers were selected. The unique codes were given one week before data collection with the help of Health Extension Workers to the selected houses of mothers (Fig. 1).

### Study variables

Dependent Variable: Late Postpartum Depression.

### Independent variable

#### Socio-demographic factors

age of the mother, age of the male partner, educational level of the mother, educational level of the male partner, occupation of the male partner, occupation of the mother, household wealth index.

#### Couples substance use and behavior

male partner alcohol use, male partner Khat use, maternal alcohol use, male partner smoking, and male partner physical fight history.

#### Reproductive characteristics-related factors

partner index of pregnancy during the current baby, the length of the inter-delivery interval, and the number of live children.

#### Maternal social and personal related factors

Intimate Partner Violence, Maternal Social Support Level, Partner Dissatisfaction with the Gender of the Current Baby, and Partner Dominance in Making Decisions.

### Operational definition

#### Late postpartum depression

is defined as depressive symptoms that occur beyond the early postpartum period, which can last up to twelve months after childbirth[33, 36]. The women who had an Edinburgh postnatal depression score of greater than or equal to thirteen during their first year after childbirth had depression and were coded as “1,“ while those who scored less than thirteen were not considered to have depression symptoms and were coded as “0” [37]. The early postpartum period was defined as the time from the birth of the baby to six weeks after birth [38].

#### Partner index of pregnancy

the male partner’s intention to have a child before pregnancy [39].

#### Postpartum intimate partner violence

those mothers who faced at least one type of intimate partner violence during their first year after childbirth among sexual, physical and psychological violence are considered to be victims of intimate partner violence [40].

#### Maternal social support

As assessed by the maternity social support scale, mothers who have MSS less than 18 have low social support, those with MSS 18–23 have medium support, and those with MSS 24–30 have high social support [41].

#### Length of the inter-delivery interval

the mother who gave birth less than two years before the preceding pregnancy is considered to have a short inter-delivery interval, and greater than or equal to two years is considered to be a normal inter-delivery interval [42].

**The Household Wealth Index**: is assessed by 38 items based on the selected ownership of assets and properties that are owned by the household. Then principal component analysis was done and divided into three equal parts: poor, which was labeled as “1,“ medium, labeled as “2,“ and rich, labeled as “3” for the purpose of this study[43].

### Data Collection tool and methods

#### Data collection tool

A face-to-face interviewer-administered structured questionnaire was used to collect the data. A validated Edinburgh postnatal depression tool that was validated in Ethiopia and has ten questions with a minimum score of zero and a maximum score of three for each item was used to measure the outcome variable. The Edinburg postnatal depression tool has a minimum score of zero and a maximum score of thirty [44]. The cut-off value of thirteen was used because it was most commonly used during pregnancy and the postpartum period, with sensitivity and specificity of 66% and 95%, respectively [45]. The tool to assess intimate partner violence was developed from related literature that has five types of intimate partner violence, a total of twenty questions, and a Cronbach’s alpha test of 0.88 [40, 46].

In addition, maternal social support was assessed by a validated maternity social support scale that has six questions with a minimum and maximum score of one and five, respectively, for each item. The tool has a minimum score of six and a maximum score of thirty [47]. The household wealth index assessment tool was developed from related literature that has 38 items containing ownership of household assets and resources. The household wealth index was classified into three categories: poor, medium, and rich [48].

Data collection and supervision were carried out by six trained married female midwifery health professionals and four trained female public health officers, respectively. Before actual data collection, training was given to data collectors and supervisors on how to collect the data on sensitive issues like “difficulty meeting husband sexual needs and intimate partner violence”. In addition, married female data collectors were recruited to overcome social norms related to sensitive issues during the data collection process.

The data were collected in the area free from family members and other persons to maintain the confidentiality of the information during the data collection process after they were informed of the purpose of the study and the nature of the topic, and written informed consent was obtained from each participant after they understood about the study and its significance. Those mothers who were busy and not present during the first contact were visited another day within the data collection period for a minimum of three episodes. Those who were identified as having depression symptoms were counseled by the data collectors and supervisors at the end of the data collection to visit nearby health institutions for possible communication with health professionals.

### Data Quality Control and Assurance

Before the actual data collection, the tool was translated from the English language to the Amharic version, which was then retranslated into English. To assure the quality of the data, a pre-test was done on 10% of the sample in Wolayita Sodo town. Initially, training was given to data collectors as well as supervisors on the questionnaires and the data collection process. Supervision was held by supervisors daily to ensure the clarity, accuracy, and consistency of the collected data on a daily basis.

### Data processing and analysis

Data were cleaned, coded, and entered into Epi Data version 3.1software before being exported to SPSS version 25 software for further analysis. Descriptive statistics such as frequency, percentages, and summary measures were carried out, and the results were presented using narrative form and tables. Both the crude odds ratio and adjusted odds ratio with a 95% confidence interval were calculated to see the association and strength of the association, respectively.

Variables with a p-value < 0.25 in the bivariate analysis were transferred to multi-variable logistic regression model to control the effect of confounders. Lastly, in multivariable analysis, variables with a p-value of < 0.05 were declared statistically significant factors. The Hosmer-Lemeshow goodness of fit test was above the level of significance. A multi-colinearity test was checked by using the variance inflation factor and colinearity statistics. Then the final results were presented in table, figure, and narrative form.

## Results

Of 479 mothers, 470 of them were participated in the study, making the response rate 98.1%. The mean age and standard deviation of the study participants were 28.82 ± 6.10 SD, respectively, and the mean age and standard deviation of the male partners were 34.73 ± 6.20 SD, respectively. This study identified 48.3% of the study participants and 59.1% of their partners as having secondary education or above. In addition, 39.6% of mothers were house wife, and 53% of their partners were merchants. Of the total study participants, 33.2% and 35.1% were at medium and rich economic levels, respectively, while the rest were in poor economic conditions (Table 1).

**Table 1:** Socio-demographic characteristics of the study participants to assess the prevalence of late postpartum depression and associated factors in Arba Minch town, Gamo zone Southern Ethiopia, 2022

Variables	Categories	Frequency	Percentage (100%)
Maternal Age	18–24	107	23.00
	25–30	201	43.00
	31–35	64	13.60
	36–40	73	15.50
	41–45	23	4.90
Husband Age	18–24	13	3.00
	25–30	140	29.80
	31–35	80	17.00
	36–40	143	30.40
	≥ 41	94	19.80
Religion	Orthodox	194	41.30
	Protestant	190	40.40
	Muslim	80	17.00
	Others*	6	1.30
Ethnicity	Gamo	216	46.00
	Gofa	88	18.70
	Amhara	94	20.00
	Oromo	48	10.20
	Others**	24	5.10
Household Wealth Index	Poor	156	33.20
	Medium	165	35.10
	Rich	149	31.70
Maternal Occupation	House wife	186	39.60
	Merchant	120	25.50
	Government employ	93	19.80
	Daily worker	29	6.20
	Student	38	8.10
	Others***	4	0.90
Partner Occupation	Merchant	249	53.00
	Government Employee	136	28.90
	Daily Worker	47	10.00
	Others****	38	8.10
Maternal Educational Level	No formal education	68	14.50
	Primary Education	175	37.20
	Secondary and above	227	48.30
Partner Educational Level	No formal education	43	9.10
	Primary Education	149	31.70
	Secondary and above	278	59.10

**Footnote**: * - Catholic, Jehovah; ** - Silte, Derashe, Konso; *** - Fisher, **** - Fisher, Carpenter, farmer

### Couples substance use and behavior related factors

Our study revealed that 20.2% and 35.1% of study participants and their partners were alcohol users, respectively. In addition, 21.3% of the study participants’ couples were Khat users, and 14% of them had a history of physical fights with other men or neighbors (Table 2).

**Table 2:** Couples substance use behavior related factors to assess the prevalence of late postpartum depression and associated factors in Arba Minch town, Gamo zone, Southern Ethiopia, 2022

Variables	Categories	Frequency	Percentage (100%)
Partner Alcohol Use	Yes	165	35.10
	No	305	64.90
Maternal Alcohol Use	Yes	95	20.20
	No	375	79.80
Partner Khat Use	Yes	100	21.30
	No	370	78.70
Partner physical fight behavior	Yes	66	14.00
	No	404	86.00

### Reproductive characteristics of the study participants

This study identified that 79.4% of the study participants have three or fewer children, while the rest have four or more children, and 69.4% of the study participants’ partners were satisfied with the gender of their current baby. In addition, 61.3% of the study participants gave birth to their current baby at the recommended range of inter-delivery intervals (Table 3).

**Table 3:** Reproductive history of the study participants related factors to assess the prevalence of late postpartum depression and associated factors in Arba Minch town, Gamo zone, Southern Ethiopia, 2022

Variables	Categories	Frequency	Percentage (100%)
Number of alive children	≤ 3	373	79.40
	≥ 4	97	20.60
Partner index of pregnancy during current baby	No	234	49.80
	Yes	236	50.20
Sex of current baby	Male	265	56.40
	Female	204	43.40
Difficulty to meet couple sexual need after childbirth	Yes	137	34.10
	No	325	58.90
Length of inter-delivery interval	Short	182	38.70
	Normal	288	61.30

### Maternal personnel and social related factors

This study revealed that 47.2% and 26.6% of household affairs were decided by the husband and the study participants, respectively. According to the findings of this study, 39.6% of the mothers received low social support during their postpartum period, and 42.6% of them suffered from postpartum intimate partner violence. In addition, 30.4% of their male partners were disappointed with the gender of their baby (Table 4).

**Table 4:** Maternal personal and social related factors to assess the prevalence of late postpartum depression and associated factors in Arba Minch town Gamo zone Southern Ethiopia, 2022

Variables	Categories	Frequency	Percentage (100%)
Husband Dominance in decision Making	Husband	222	47.20
	Jointly	123	26.20
	Wife	125	26.60
Maternal Social Support	Low	75	15.97
	Medium	111	23.63
	High	284	60.40
Partner satisfaction to gender of current	No	143	30.40
	Yes	326	69.40
Intimate partner violence during postpartum	No	270	57.40
	Yes	200	42.60

### Prevalence of late Postpartum Depression

**Fig. 2:**
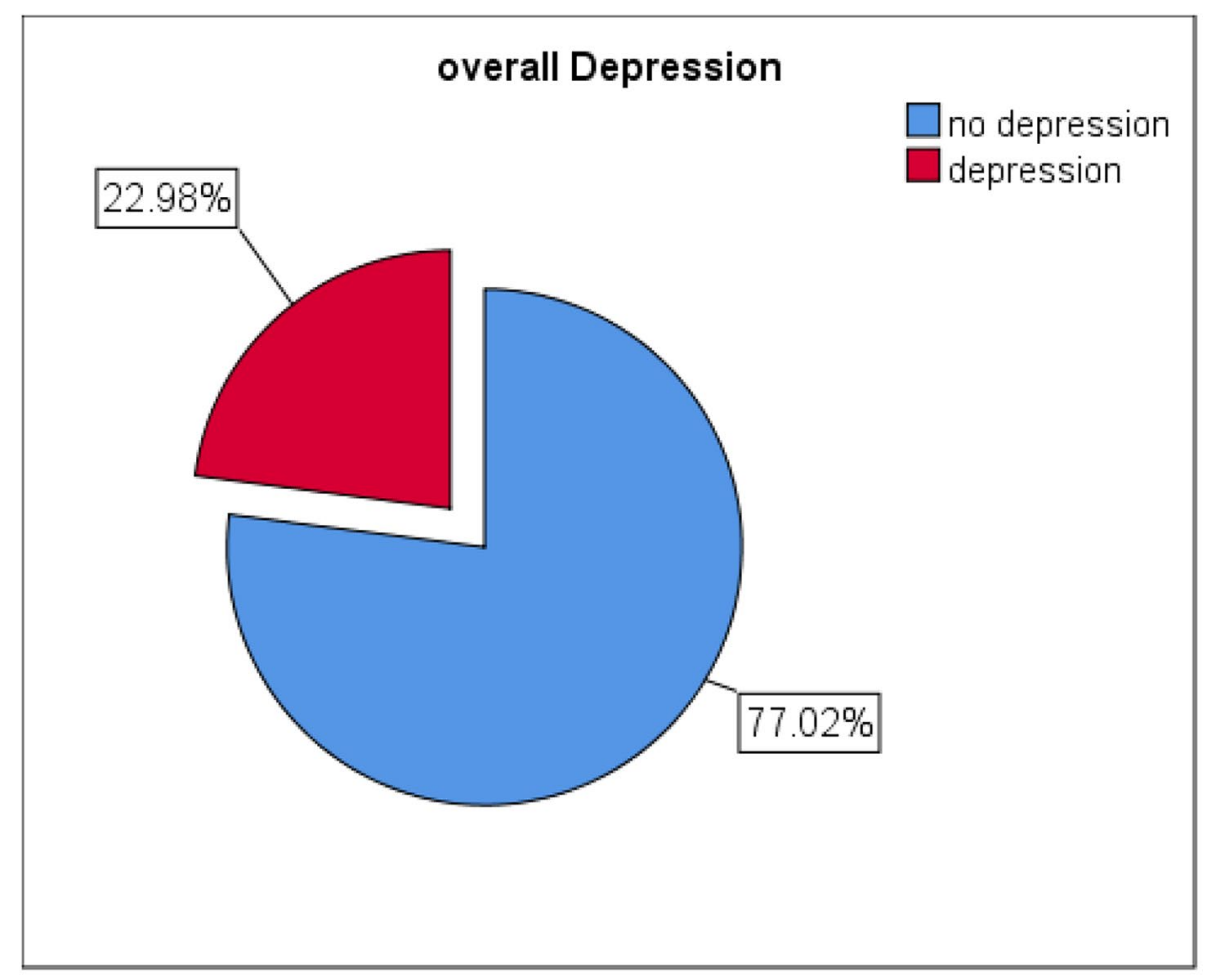
Overall Prevalence of postpartum depression in Arba Minch town, Gamo zone, Southern Ethiopia, 2022

The findings of our study showed that the prevalence of late postpartum depression is 22.98% (95% CI: 19.16 to 26.80) (Fig. 2).

### Factors Associated with late Postpartum Depression in Arba Minch town, Gamo Zone, Southern Ethiopia, 2022

In bivariate analysis, eleven variables were significantly associated with late postpartum depression, and in multivariable analysis, six variables were significantly associated with late postpartum depression after the effect of confounders was controlled. Maternal and partner educational level, household wealth index, partner index of pregnancy during the current baby, husband dissatisfaction with the gender of the current baby, husband alcohol and Khat use, maternal social support level, postpartum intimate partner violence, difficulty meeting the husband’s sexual need after childbirth, and length of the inter-delivery interval were significantly associated factors with late postpartum depression in bivariate analysis.

In multivariable analysis, husband dissatisfaction with the gender of the current baby, difficulty meeting the husband’s sexual need after childbirth, length of the inter-delivery interval, husband Khat use, postpartum intimate partner violence, and maternal social support level were significantly associated factors with late postpartum depression at a p-value of < 0.05.

This study revealed that the odds of late postpartum depression among mothers whose husband uses Khat were 2.64 times higher than those whose partners never use Khat (AOR = 2.64; 95% CI: 1.18, 5.91). Similarly, the odds of late postpartum depression among mothers whose partners were dissatisfied with the gender of their current baby were 2.53 times higher than those whose partners were satisfied with the gender of their infant (AOR = 2.53; 95% CI: 1.22, 5.24).

The odds of late postpartum depression among mothers whose delivery interval was short were 6.80 times higher than those of mothers who were in a normal inter-delivery interval (AOR = 6.80; 95% CI: 3.34, 13.84). In addition, the odds of late postpartum depression among mothers who faced difficulty meeting their husband’s sexual needs during the postpartum period were 3.21 times higher than their counterparts (AOR = 3.21; 95% CI: 1.62, 6.37).

Furthermore, the odds of late postpartum depression among mothers who have faced IPV during the postpartum period were 4.08 times higher than those who had not experienced it after childbirth (AOR = 4.08; 95% CI: 1.95, 8.54), and the odds of this problem were 2.50 times higher in mothers with low social support when we compared with those who have high social support (AOR = 2.50; 95% CI: 1.25, 4.50) (Table 5).

**Table 5:** Factors associated with late postpartum depression in Arba Minch town Gamo zone Southern Ethiopia, 2022

Variables	Categories	Late postpartum depression		COR(95%CI)	AOR(95%CI)
		Yes(108)	No(362)		
Wealth Index	Poor	52(33.3%)	104(66.7%)	2.73(1.57, 4.77)	1.74(0.72,4.22)
	Medium	33(20.0%)	132(80%)	1.37(0.76, 2.46)	0.82(0.33,2.01))
	Rich	23(15.4%)	126(84.6%)	1	1
Maternal Educational Status	No formal education	13(19.1%)	55(80.9%)	1.00(0.50,1.99)	0.63(0.20,1.94)
	Primary education	51(29.1%)	124(70.9%)	1.70(1.07,2.70)	1.06(0.05,2.24)
	Secondary and above	44(19.4%)	183(80.6%)	1	1
Husband educational status	No formal education	22(51.2%)	21(48.8%)	4.55(2.33,8.90)	2.01(0.72, 5.64)
	Primary education	34(22.8%)	115(77.2%)	1.28(0.79,2.09)	0.98(0. 43,2.20)
	Secondary and above	52(18.7%)	22(81.3%)	1	1
Partner index of pregnancy during current baby	No	76(32.5%)	159(67.5%)	3.05(1.92, 4.84)	1.37(0.65,2.89)
	Yes	32(13.6%)	203(86.4%)	1	1
Husband satisfaction with gender of current baby	No	59(41.3%)	85(58.7%)	3.97(2.53, 6.23)	2.53(1.22,5.24)*
	Yes	49(15%)	277(85%)	1	1
Husband Alcohol use	Yes	59(35.8%)	106(64.2%)	2.84(1.80,4.50)	1.74(0.86,3.50)
	No	49(16.1%)	256(83.9%)	1	1
Husband Khat use		33(33%)	67(67%)	1.94(1.19,3.15)	2.64(1.18,5.91)*
		75(20.3%)	295(79.7%)	1	1
Maternal social support level	Low	75(40.3%)	111(59.7%)	4.70(3.05,8.09)	2.50(1.25,4.50)*
	High	33(11.6%)	251(88.4%)	1	1
Postpartum IPV	No	25(10%)	226(90%)	1	1
	Yes	83(37.9%)	136(62.1%)	5.52(3.36, 9.05)	4.08(1.95,8.54)*
Length of inter-delivery interval	Less than two years	81(40.1%)	121(59.9%)	5.36(3.25,8.86)	6.80(3.34,13.84)*
	Two years or more	27(10.1%)	241(89.9%)	1	1
Difficulty to meet couple sexual need	Yes	49(35.8%)	88(64.2%)	4.90(2.89,8.33)	3.21(1.62,6.37)*
	No	27(10.2%)	238(89.8%)	1	1

**Footnote**:* Significantly associated factors at p < 0.05, COR – Crude Odds Ratio; AOR – Adjusted Odds Ratio, 1 – reference group; **IPV**: Intimate Partner Violence

## Discussion

Generally, this study assessed the prevalence of late postpartum depression and associated factors in Arba Minch town. As identified by this study, 22.98% of mothers suffered from late postpartum depression. Partner Khat use, maternal social support, intimate partner violence during the postpartum period, partner dissatisfaction with the gender of the current baby, difficulty in meeting partner sexual needs after childbirth, and the length of the inter-delivery interval were significantly associated factors with late postpartum depression.

This study revealed the prevalence of late postpartum depression was 22.98%, which is in line with the findings of a study conducted in the Awi Zone, Ethiopia 23.7% [35]. This might be due to the similarity in socio-demographic characteristics among the study participants. However, the finding of our study was higher than the finding of the study conducted in Ghana16.8% [49]. The possible justification might be the study conducted in Ghana has used small sample size that can leads to the underestimation of the prevalence LPPD. Furthermore, it was lower than the cross-sectional studies conducted in Eastern Turkey 34.6%[50], and Kazakhstan59.4% [51]. This might be due to the difference in socio-economic status among the study participants, as those mothers in low-income countries may not perceive it as a problem and be less likely to disclose it during data collection, which might underestimate the problem in this study setting.

The partner Khat use was a significantly associated factor with late postpartum depression, which was supported by the evidence obtained from the studies conducted in Ethiopia [52, 53]. This might be because substance users might not take care of their infant and wife, which leads to family maltreatment as a result of substance effects, as the physical and emotional demands of the mother that increase during the postpartum period need family support. This justification is supported by evidence obtained from a spouse’s substance use and family maltreatment [54]. In addition, being a mother is a stressful time in the life of reproductive-age women that requires emotional and psychological support from the male partner and family members for the sake of maternal mental health during the postpartum period.

Intimate partner violence during the postpartum period is significantly associated with late postpartum depression, as supported by evidence obtained from a systematic review conducted in Ethiopia [52], and a study conducted in the United States of America on the co-occurrence of postpartum depression and intimate partner violence [55]. This might be because those mothers who suffered from intimate partner violence during the postpartum period might be at higher risk of feeling lonely and becoming hopeless, which might push them to develop postpartum depression.

The maternal social support level was a significantly associated factor with late postpartum depression. This is corroborated by the studies conducted in the Awi Zone of Ethiopia [35], and Iran [56]. This might be during the postpartum period when mothers become too busy to give care for their husbands, infants, and other family members, and if they have not gotten extra support from their husbands and other family members, they become stressed and hopeless, which might lead to postpartum depression.

In addition, partner dissatisfaction with the gender of the current infant was a significantly associated factor with late-postpartum depression. This is supported by the evidence obtained from the findings of the study conducted in the Awi Zone of Ethiopia [35], and by the meta-analysis carried out in China [57]. This might be because those dads who were not satisfied with the gender of their infant might demoralize their wives and not need to care for their infant, which leads to postpartum depression.

The difficulty in meeting the couple’s sexual needs was a significantly associated factor with late postpartum depression, which was supported by a study conducted on maternal depressive symptoms and sexual distress trajectories during the first year after childbirth [58]. This might be because, during the postnatal period, mothers might be at higher risk of experiencing pain during sexual intercourse due to the effect of hormonal changes as a result of lactation. In addition, during the postpartum period, there might be a recovery of the birth canal from childbirth injuries that leads to painful intercourse that result in sexual stress among mothers, which affects maternal mental health and can result in a loss of confidence among those mothers.

Similarly, this study identified the length of the inter-delivery interval as a significantly associated factor with late postpartum depression, but it was not replicable to our knowledge, as there is no literature that supports these findings. However, the study conducted in Hartford revealed that anemia is significantly associated with postpartum depression, and the major cause of anemia in postpartum women is a short inter-delivery interval [59]. In addition, mothers with short inter-delivery intervals were at higher risk of work overload to care for infants who need more maternal care that might leads to postpartum depression. Furthermore, mothers with short inter-delivery intervals were at higher risk for obstetric complications that lead to maternal weakness, which leads to mental health problems if she is unable to care for their infant and husband during the postpartum that might leads depression.

### Limitation and strength of the study

As this was a cross-sectional study, it does not identify a cause-and-effect relationship. In addition, there was a scarcity of literature regarding this topic, especially in low-income countries. The strength of this study was that it focused on a neglected area of study, and furthermore, the household’s wealth index was assessed.

## Conclusions

Generally, this study indicates that the prevalence of late postpartum depression is high in this study setting. Husband Khat use, husband dissatisfaction with the gender of the current baby, difficulty in meeting couples’ sexual needs after childbirth, length of the inter-delivery interval, postpartum intimate partner violence, and low maternal social support were significantly associated factors with LPPD.

### Recommendations

Therefore, the Ministry of Health, Zonal Health Departments, and non-government organizations should develop effective strategies to overcome late postpartum depression based on identified factors. In addition, screening for PPD should be integrated into the expanded program for immunization. Furthermore, screening for postpartum intimate partner violence and maternal social support level should be linked with maternity and child health care services. Finally, partner preference for a particular gender and women with short inter-delivery intervals should be identified by community health extension workers and counseled. In addition, it is better if it is focused on this population category with a strong study design by other researchers.

## Data Availability

The data used for this study is available upon reasonable request from the corresponding author at any time if required through (gmail: lemafika2014@gmail.com).

## Abbreviations

PPIV: Postpartum Intimate Partner Violence
LPPD: Late Postpartum Depression
